# Two-Stage Crystallization Combining Direct Succinimide Synthesis for the Recovery of Succinic Acid From Fermentation Broth

**DOI:** 10.3389/fbioe.2019.00471

**Published:** 2020-01-15

**Authors:** Yiwen Xiao, Zhibin Zhang, Ya Wang, Boliang Gao, Jun Chang, Du Zhu

**Affiliations:** ^1^Key Laboratory of Protection and Utilization of Subtropic Plant Resources of Jiangxi Province, Jiangxi Normal University, Nanchang, China; ^2^Key Laboratory of Bioprocess Engineering of Jiangxi Province, College of Life Sciences, Jiangxi Science and Technology Normal University, Nanchang, China

**Keywords:** succinimide, recovery, co-crystals, succinic acid, urea

## Abstract

Succinic acid is an important chemical and raw material widely used in medicine, food, biodegradable materials, fine chemicals, and other industrial fields. However, traditional methods for purifying succinic acid from fermentation broth are costly, poorly efficient, and harmful to the environment. In this study, an efficient method for purifying succinic acid from the fermentation broth of *Escherichia coli* NZN111 was developed through crystallization and co-crystallization with urea. First, the filtrate was collected by filtering the fermentation broth, and pH was adjusted to 2.0 by supplementing sulfuric acid. Crystallization was carried out at 8°C for 4 h to obtain succinic acid crystals. The recovery rate and purity of succinic acid were 73.4% and over 99%, respectively. Then, urea was added to the remaining solution with a mass ratio of urea to residual succinic acid of 4:1 (*m*_*urea*_/*m*_*SA*_). The second crystallization was carried out at pH 2 and 4°C for 12 h to obtain succinic acid-urea co-crystal. The recovery rate of succinic acid residue was 92.0%. The succinic acid-urea crystal was further mixed with phosphorous acid (4.2% of the mass of succinic acid co-crystal) and maintained at 195°C for 6 h to synthesize succinimide, and the yield was >80%. This novel and efficient purification process was characterized by the significantly reduced urea consumption, and high succinic acid recovery (totally 95%), and high succinimide synthesis yield (80%). Thus, this study potentially provided a novel and efficient strategy for the industrial production of succinic acid and succinimide.

## Introduction

Succinic acid is an important renewable four-carbon building block chemical that is prevalent in humans, animals, plants, and microorganisms (Li et al., 2010; Alexandri et al., 2019). Succinic acid has been widely used in medicine, food, the chemical industry, and agriculture (Delhomme et al., 2009). Presently, the market demand for succinic acid has increased dramatically owing to its application in green solvents and biodegradable polymers (Jansen and van Gulik, 2014). Succinic acid is primarily produced via chemical synthesis using maleic anhydride as a raw material, which causes serious environmental and economic concerns such as increased pollution and energy consumption, thereby rendering this process unsustainable (Cukalovic and Stevens, 2008; Pinazo et al., 2015). Thus, a novel and green strategy for producing succinic acid through the biotransformation of cheap, renewable materials has been suggested (Sheldon and Sanders, 2015). For example, succinic acid can be produced via the fermentation of cassava, straw, and waste biomass by microorganisms, such as engineered *Escherichia coli* (Zhang et al., 2018), *Actinobacillus succinogenes* (Gunnarsson et al., 2014; Dessie et al., 2018), and *Anaerobiospirillum succiniciproducens* (Akhtar et al., 2013; Lee et al., 2015), respectively. However, an efficient method of purifying succinic acid from these fermentation broths remains unavailable.

Generally, the bio-production of succinic acid involves four steps: right raw material selection, pretreatment, fermentation, and purification. The separation and purification procedures account for 50–80% of the total production costs (Cheng et al., 2012; López-Garzón and Straathof, 2014). Therefore, the costs involved in these steps must be reduced to promote the industrialization of biologically synthesized succinic acid. Conventional methods for separating succinic acid from the fermentation broth include calcium salt precipitation, solvent extraction, ion exchange, electrodialysis, and membrane separation (Khunnonkwao et al., 2018; Prochaska et al., 2018; Antczak et al., 2019). However, these methods have many disadvantages, including high cost, low efficiency, and environmentally hazardous elements, which impede their use in industrial applications. For example, the yield of isolated succinic acid using calcium salt precipitation is only 52% (Li et al., 2010); meanwhile, a large amount of calcium sulfate waste is produced. Succinic acid can also be separated using a two-phase system. This process is simple and energy saving but cannot be used to remove heteroacids; therefore, a large amount of solvent is wasted (Matsumoto and Tatsumi, 2018). Previous reports have shown that increasing the yield of succinic acid from residual solvent is difficult, costly, and polluting. Thus, a strategy is required to transform the succinic acid that is available in residues solvent into an easily purified and valuable product, such as succinimide (Deng et al., 2014), Diethyl succinate (Kolah et al., 2008; López-Garzón et al., 2012; Orjuela et al., 2012), poly (butylene succinate), γ-butyrolactone (Hong et al., 2012a), and tetrahydrofuran (Hong et al., 2012b). Amongst them, succinimide is an important intermediate used as for medicine, pesticides, and silver-plating industry (Liu et al., 2018). Therefore, a simple, highly efficient, and green method for separating succinic acid from a microorganism fermentation broth is needed.

In the present study, a simple and inexpensive method involving crystallization and urea co-crystallization was developed to separate succinic acid from fermentation broth efficiently. This study also focused on the feasibility of succinimide synthesis following succinic acid–urea co-crystallization. The established method is not only simple and highly efficient in separating succinic acid with high purity and yield, but also selective for succinic acid among various carboxylic acids. More importantly, the method provides a novel strategy for the full recovery of residual succinic acid following the purification process.

## Materials and Methods

### Materials

Succinic acid and succinimide standards were purchased from Sigma-Aldrich (Shanghai, China). Urea, phosphorous acid, pyruvic acid, and acetic acid were acquired from Aladdin (Shanghai, China). Methanol and acetonitrile were HPLC graded and acquired from Merck (Darmstadt, Germany). Actual succinic acid fermentation broth was provided by Professor Zhimin Li (East China University of Science and Technology). The glucose standard was procured from Xilong Chemical Co., Ltd. (Guangzhou, China).

The succinic acid fermentation broth was used as a raw material in accordance with previously described methods (Chen et al., 2014). The concentrations of succinic acid, acetic acid, pyruvic acid, glucose, and protein were 106.17, 3.37, 0.99, 4.15, and 0.043 g/L, respectively.

### Pretreatment

The broth was centrifuged at 8,000 rpm for 30 min to remove bacteria, macromolecular impurities, and insoluble substances. Then, activated carbon was added to decolorize the broth under 30°C, pH 6.8, and 250 rpm for 40 min.

### Crystallization

**Figure 1:**
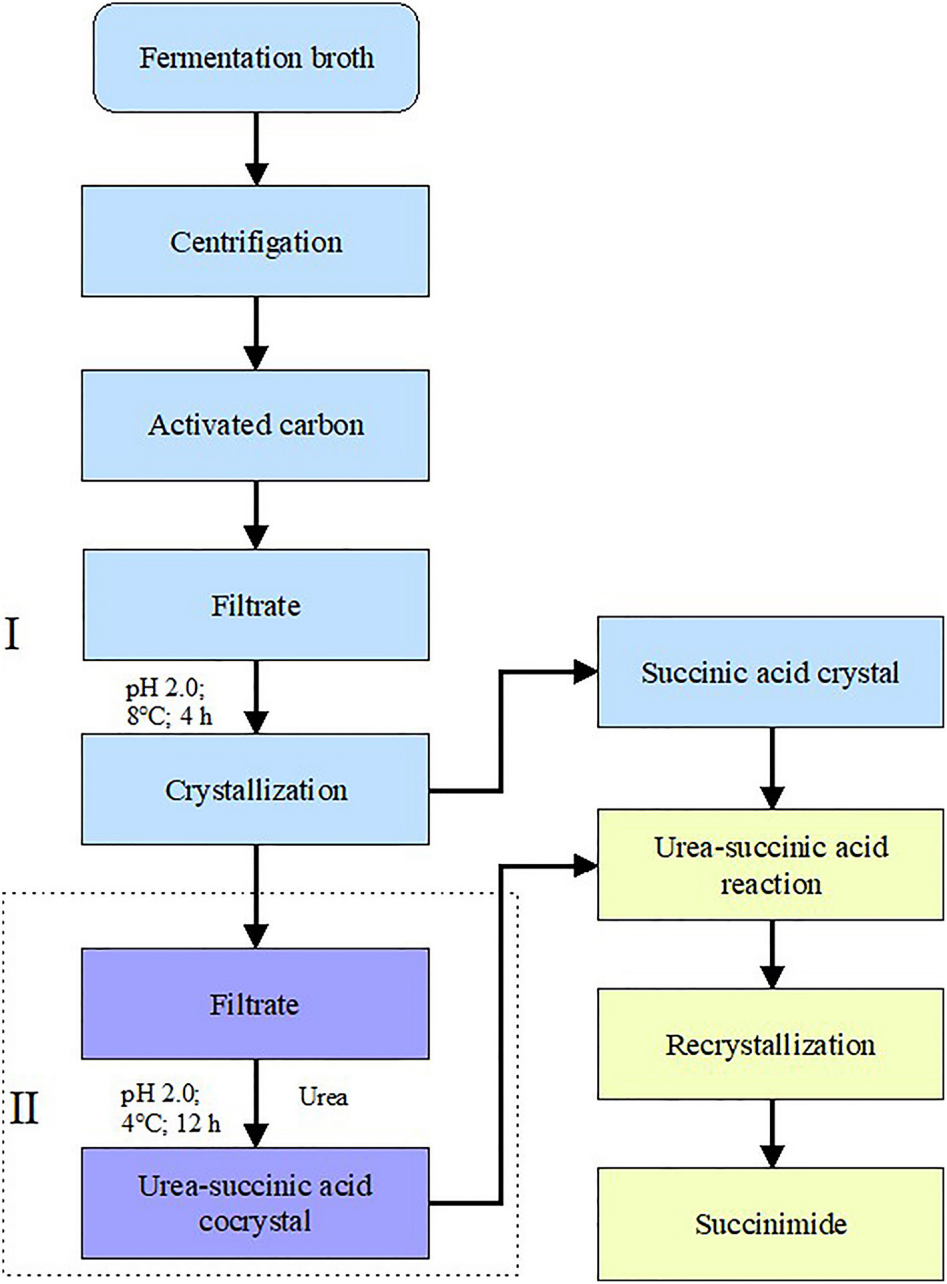
Schematic diagrams of two-stage crystallization combining direct succinimide synthesis for succinic acid recovery. (I) Direct crystallization and (II) co-crystallization of succinic acid with urea.

The schematic diagram was shown in Figure 1. At stage I, the decolorized broth was cooled and then crystallized directly at various temperatures (4, 8, 12, 16, 20, 28, 32, 36, and 40°C) and pH 2.0 for 4 h. During crystallization, the succinic acid, pyruvate, and acetic acid concentrations were determined. The broth volume was measured before and after the experiment to enable the recovery rate of succinic acid to be calculated. The experimental processes for measuring time and pH were similar to for measuring temperature.

At stage II, after direct crystallization, the pH of the broth was adjusted to 2.0. Then, urea was added to 50 mL of the broth at mass ratios of urea to succinic acid of 1:1, 2:1, 3:1, 4:1, 5:1, and 6:1, respectively. The mixtures were maintained at 4°C for 12 h for crystallization to occur. The concentration and the recovery rate of succinic acid were measured using ultra-performance liquid chromatography UPLC (Acquity H-class, Waters, USA).

### Succinimide Synthesis

In this procedure, 11.81 g of succinic acid, 3.07 g of urea, 10 mL of water, and 1.0 g phosphite solid were mixed and dissolved at 80°C. Succinimide was synthesized at 195°C for 5 h. The reaction system was cooled to 80°C, and 8 mL of water and 1 g of activated carbon were added. Thereafter, the mixture was filtered with hot water, and crystallized at 25°C for 12 h to obtain crude succinimide, which was further recrystallized with ethanol. The effects of the mass ratio of succinic acid to urea, reaction temperature, reaction time, and amount of catalyst on the reaction process were investigated on the basis of the experimental operation above. Succinimide was synthesized with succinic acid-urea co-crystals under optimal reaction conditions. The mass ratio of succinic acid to urea was adjusted to 2:1 using the condensed succinic acid as a raw material, adding ~4.2% solid phosphorite, stirring, heating to 195°C, and reacting at a constant temperature for 6 h. The succinimide yield was subsequently calculated.

All experiments were conducted in triplicate.

### Methods of Analysis

Organic acids were analyzed using UPLC equipped with an Acquity UPLC BEH, a C_18_, and 1.7 μm, 2.1 × 100 mm chromatography column (Waters, USA). The mobile phase consisted of acetonitrile and 3 mM H_2_SO_4_ (CH_3_CN:3 mM H_2_SO_4_ = 3:97, v/v). The flow rate was 0.2 mL/min, and the wavelength was set at 210 nm. Protein contents were determined using the Bradford (Bradford, 1976), with bovine serum albumin (Sigma Chemical Co.) used as a standard. Yield and purity were calculated as followed:

Yield (%) = [dry weight of succinic acid in the recovered crystals (g)/weight of succinic acid in the initial fermentation broth (g)] × 100%.
Purity (%) = [dry weight of succinic acid in crystals recovered (g)/dry weight of the recovered crystals (g)] × 100%.
Succinimide yield was obtained as follows:
Succinimide yield (%) = actual product quality (g)/theoretical product quality (g) × 100%.

## Results

### Optimization of Succinic Acid Crystallization From Fermentation Broth

#### Crystallization From Simulated Fermentation Broth

**Figure 2:**
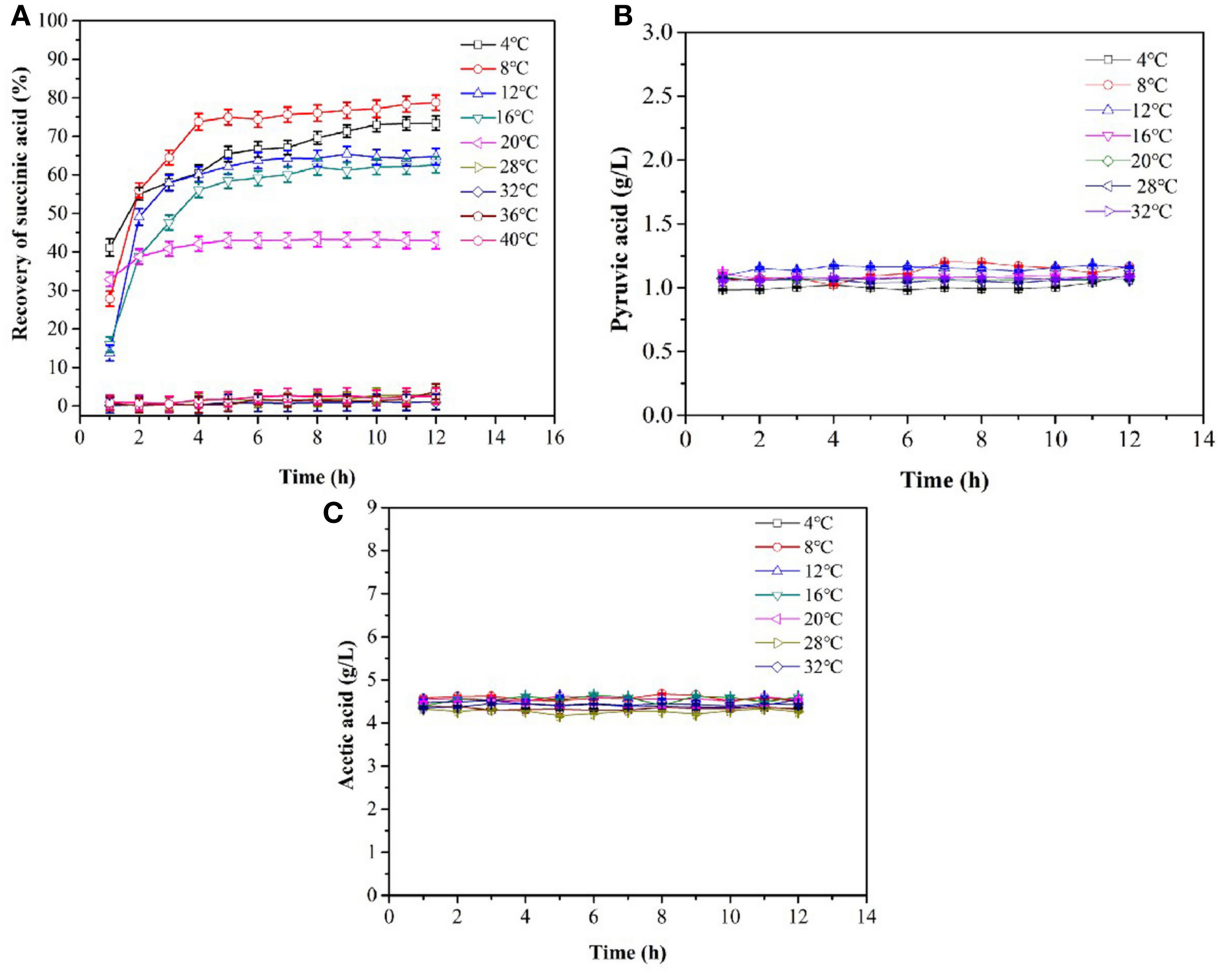
Cooling crystallization of the simulated fermentation broth. **(A)** Yield of succinic acid during the crystallization, **(B)** the concentration of pyruvic acid, **(C)** the concentration of acetic acid.

No succinic acid crystals were observed within 12 h from 32 to 40°C (Figures 2A, **4B**). The maximum amount of succinic acid crystal was obtained at 8°C. The number of crystals increased drastically within 120 min and the succinic acid yield was 55.9%. The increase in crystals yield at 20°C was not significant after 3 h. At 4°C, the crystallization increased with time, and the yield of succinic acid was stable at 73.0% after 10 h. The pyruvic acid and acetic acid concentrations remained at initial values during the whole period of crystallization (Figures 2B,C). This result indicated that pyruvic acid and acetic acid were not crystallized. Figure 2A shows that temperatures significantly affected succinic acid crystallization. The optimal temperature and time value for the crystallization of succinic acid from the fermentation broth were 8°C and 4 h, respectively. In addition, the largest amount of crystals in powder form was obtained at <20°C. Prismatic and flaky crystals would be obtained when the broth was slowly cooled at room temperature.

Impurities such as acetic acid and pyruvate could not be crystallized from the fermentation broth during crystallization (Figures 2B,C). Therefore, the influence of these impurities on the recovery rate of succinic acid could be not considered in the subsequent co-crystallization of succinic acid with urea.

**Figure 3:**
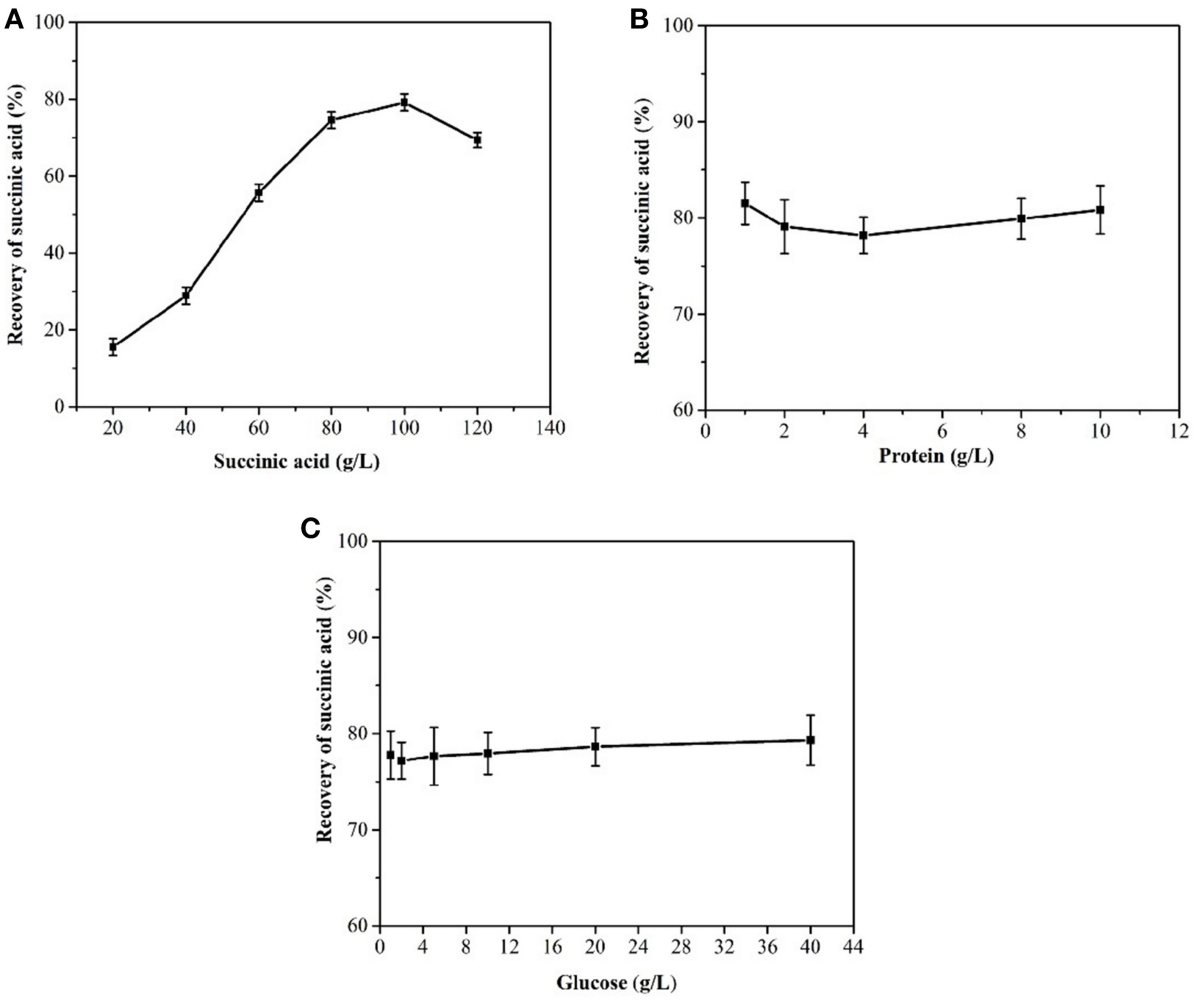
Effects of succinic acid **(A)**, protein **(B)**, and glucose concentration **(C)** on the cooling crystallization of the simulated fermentation broth.

When the succinic acid concentration of succinic acid was <100 g/L, the yield of cooling succinic acid crystallization increased as the succinic acid concentration increased (Figure 3A). Glucose and protein were noted to have no influence on the recovery rate of succinic acid (Figures 3B,C), consistent with the results of Wang et al. (2014). The average recovery rate of the succinic acid fermentation broth was above 75.0%.

#### Effect of pH on Succinic Acid Crystallization

**Figure 4:**
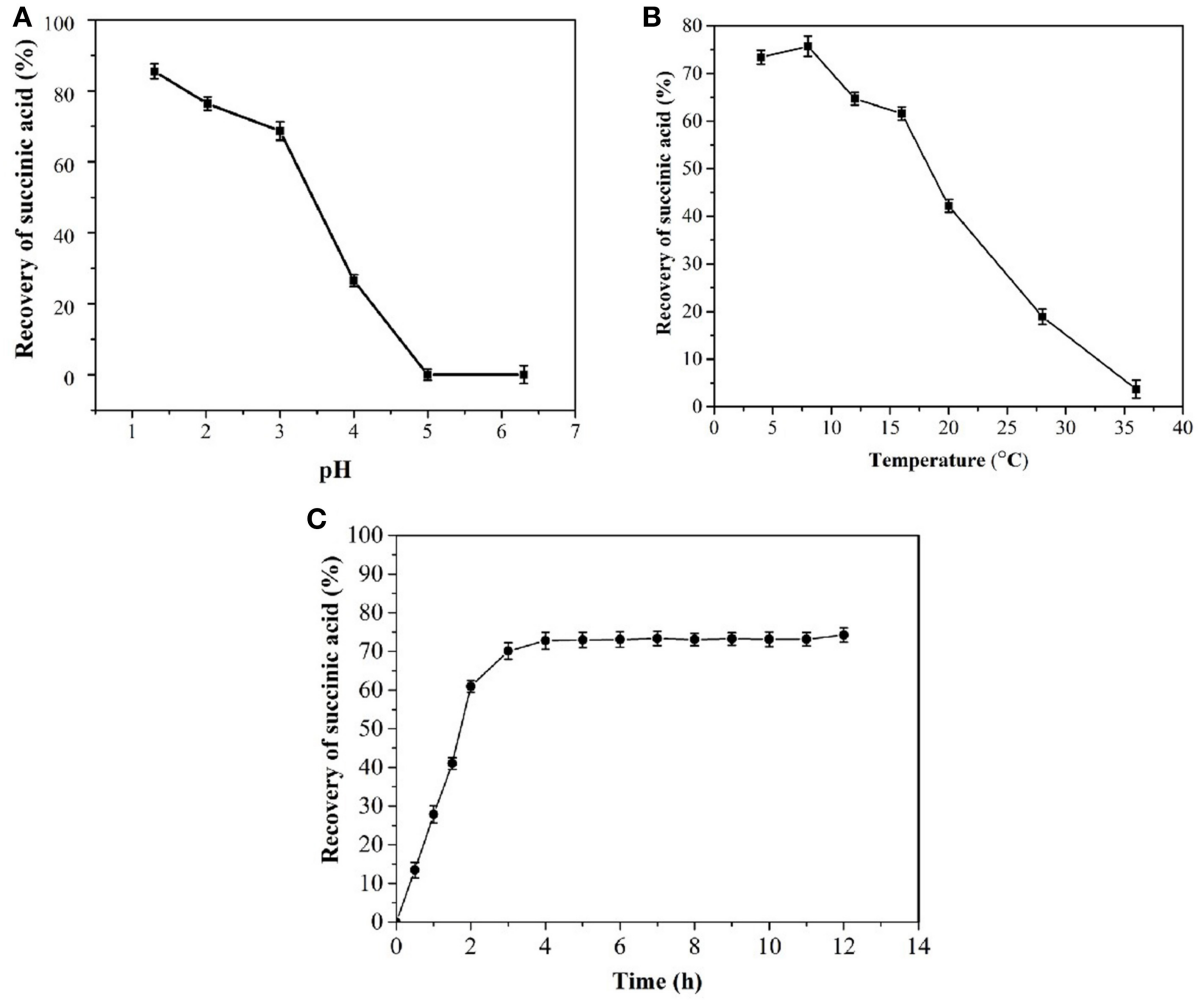
Effects of pH **(A)**, temperature **(B)**, and time **(C)** on the crystallization of succinic acid from fermentation broth.

Increases in pH lead to decreases in the succinic acid recovery rate (Figure 4A). A pH of <2 resulted in a recovery rate of >76.4% succinic acid, possibly because the compound was in a free molecular state and had low solubility at low pH. A high pH would result in the partial dissociation of succinate molecules, thereby increasing solubility. Thus, extracting succinate from a solution at pH >2.0 was difficult, leading to a decreased recovery rate.

#### Effect of Time on Succinic Acid Crystallization

Figure 4C shows that the recovery rate of succinic acid increases with time over a period of 1–2 h. Crystal growth was slowed down after 2 h and ceased after 4 h. Therefore, the optimal period for cooling and crystallization was 4 h.

#### Effect of Temperature on Succinic Acid Crystallization

When the temperature was <10°C, the recovery rate of succinic acid was >70% (Figure 4B). The recovery rate of succinic acid at 8°C was higher than that at 4°C. Possibly because the lower the temperature, the slower the molecules diffuse. This condition led to the slow crystal growth after nucleation. Therefore, the optimal temperature of succinic acid crystallization was determined to be 8°C. Following the first crystallization step, the succinic acid concentration in the broth was 28 g/L.

### Co-crystallization of Succinic Acid With Urea

#### Influence of the Mass Ratio of Urea to Succinic Acid on the Urea Co-crystallization Process

**Figure 5:**
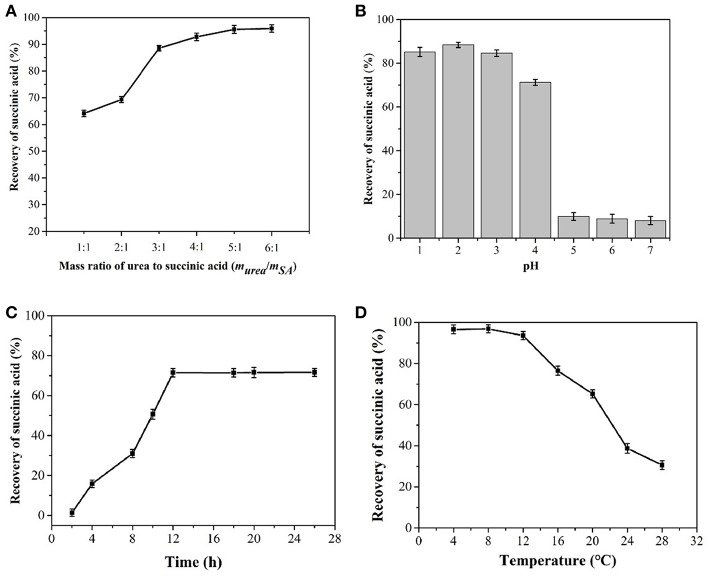
Influence of the mass ratio of urea to succinic acid **(A)**, pH **(B)**, time **(C)**, and temperature **(D)** on co-crystallization process from fermentation broth after cooling crystallization.

The influence of a range of urea to succinic acid mass ratios from 1:1 to 6:1 on the yield of succinic acid was also investigated. Figure 5A shows that no urea and succinic acid were precipitated into crystals at mass ratio of 1:1. When the ratio was increased to 2:1, 70% of succinic acid could be co-crystallized. With the addition of urea, the succinic acid content in the fermentation broth was reduced. The optimal mass ratio of urea to succinic acid was determined to be 4:1.

#### Effect of pH on Co-crystallization With Urea

The pH of the feed solution significantly affected co-crystallization with urea (Figure 5B). At pH 2.0, the largest number of co-crystallites was obtained. As succinic acid was present in ionic form in the solution, the formation of hydrogen bonds between succinic acid and urea was difficult and a pH > 5.0 resulted in low succinic acid recovery. Therefore, the optimal pH was determined to be 2.0.

#### Effect of Time on Co-crystallization With Urea

The co-crystallites of succinic acid and urea grew rapidly into nucleation within 5 h (Figure 5C) and stopped at 12 h. This result indicated that the optimal total crystallization time was 12 h.

#### Effect of Temperature on Co-crystallization With Urea

Temperature was inversely related to the number of succinic acid and urea co-crystallites (Figure 5D). The maximum number of co-crystallites was obtained at 4°C.

### Feasibility of Succinimide Synthesis

**Figure 6:**
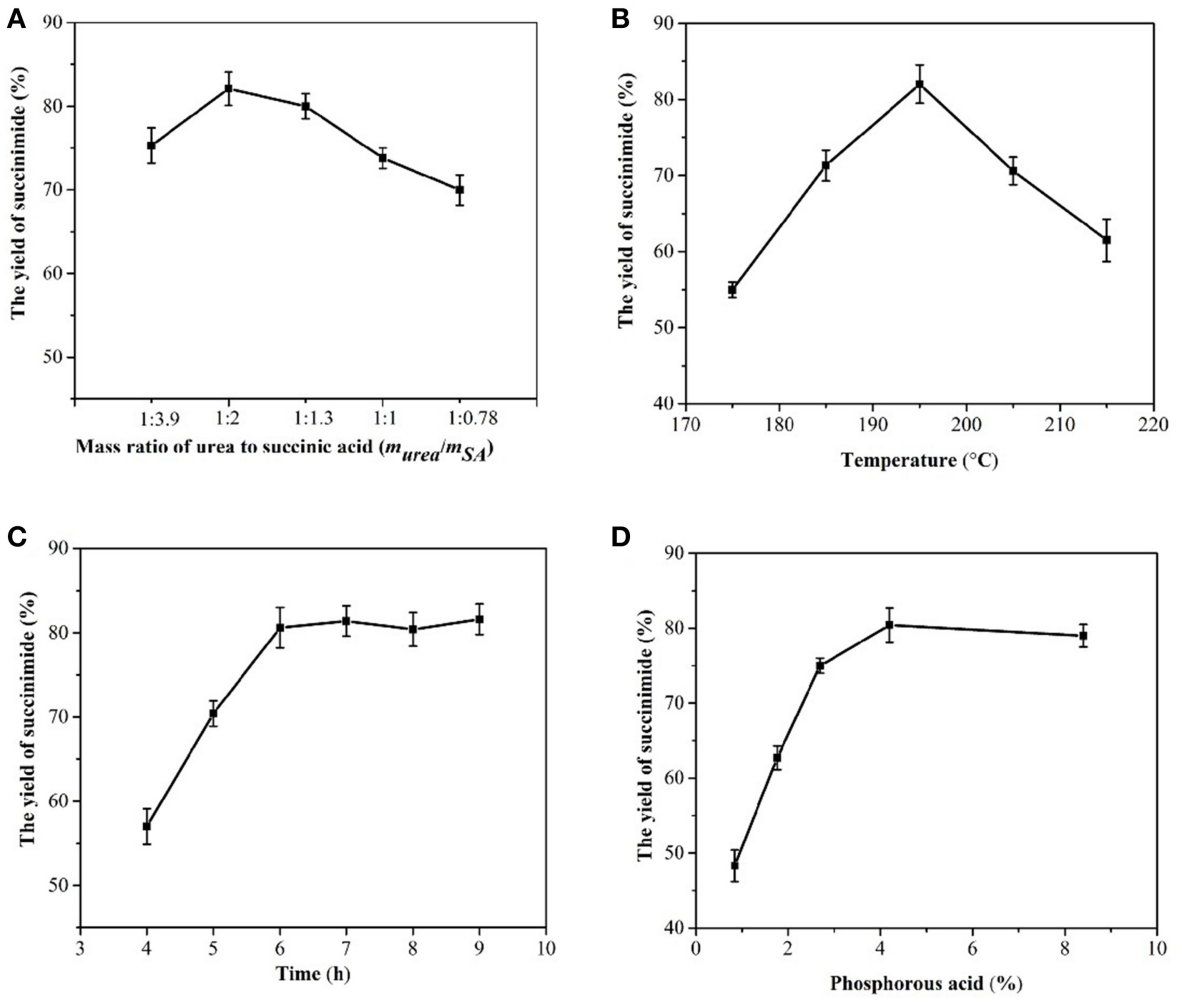
Effects of the mass ratio of succinic acid to urea **(A)**, temperature **(B)**, time **(C)**, and phosphorous acid **(D)** on succinimide yield.

At an initial mass ratio of succinic acid to urea of 3.9:1, the succinic acid was slightly excessive, and the succinimide yield was 75% (Figure 6A). In terms of cost, excess urea shifted the balance toward production. However, excessive urea, would result in the formation of the byproduct, that is, succinamide. The selected mass ratio of succinic acid to urea was therefore 2:1 (Charville et al., 2010).

The reaction of succinic acid and urea is endothermic; hence, increasing the temperature helps to shift the reaction in an endothermic direction and increase the amount of succinimide produced (Pavelka and Grmela, 2019). However, when the temperature is too high, carbonization occurs easily and energy consumption is high. In this study, the reaction temperature was 195°C (Figure 6B).

The maximum amount of succinimide was synthesized during a reaction period of 4-6 h (Figure 6C). Therefore, the optimal reaction time was determined to be 6 h, and the yield of succinimide was 80.6%.

Phosphorous acid can accelerate a reaction to reach its equilibrium and shorten the reaction time. In the reactions with 11.81 g of succinic acid and 3.07 g urea, 0.5 g phosphorous acid, e.g., 4.2% of the mass of succinic acid was found to be an appropriate addition to the reaction system (Figure 6D).

**Figure 7:**
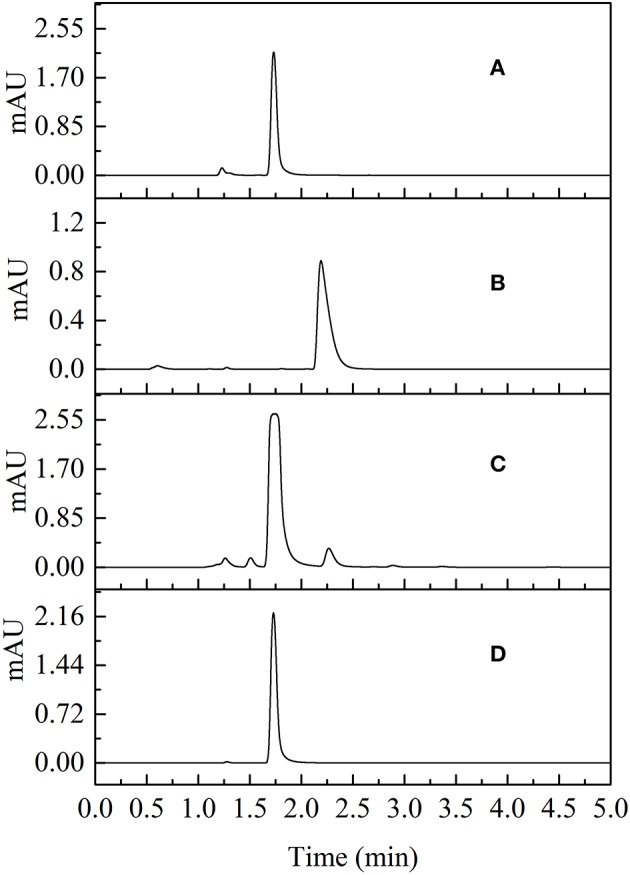
Chromatographic comparisons of succinimide standards and succinimide synthesis from succinic acid-urea samples at 210 nm (**A** succinimide standard; **B** succinic acid-urea; **C** succinimide synthesis from succinic acid-urea; **D** recrystallization of succinimide synthesis from succinic acid-urea).

The yield of 80% succinimide was obtained by adjusting the mass ratio of succinic acid and urea to 2:1 using condensed succinic acid as a raw material, adding ~4.2% solid phosphorite, stirring, heating to 195°C, and reacting at a constant temperature for 6 h. Succinimide standards and succinimide synthesized from succinic acid–urea samples were compared using UPLC. The results revealed that succinimide synthesis by succinic acid–urea co-crystallization was successful (Figure 7).

## Discussion

Using a simple process at a low cost, succinic acid was separated from a complex fermentation broth by cooling crystallization and urea co-crystallization, with a high rate of recovery. So far, other reports on crystallization have shown that cooling crystallization is usually combined with vacuum distillation, ion exchange adsorption, and salting-out extraction. Li et al. (2010) analyzed one-step crystallization and obtained yield and purity of 70% and 90%, respectively. Luque et al. (2009) applied direct vacuum distillation-crystallization, and obtained yield and purity of 75% and 97% for the simulated broth, respectively. In the present study, the yield and purity of succinic acid recovered from fermentation broth were 73.4% and 99%, respectively, which were the same as those obtained using one-step crystallization and direct vacuum distillation-crystallization, as stated above. However, the purity of succinic acid obtained using our newly defined method was better than that obtained through one-step crystallization and direct vacuum distillation-crystallization, even though the yields were almost the same. Vacuum distillation as a unit operation was required, but this process consumed high energy and the impurity of fermentation was also concentrated. Therefore, the high succinic acid titer of 106.17 g/L in this study could be helpful for crystallization.

The results obtained from other technologies are listed in Table 1. Sun et al. (2014) developed salting-out extraction combined with crystallization to recover succinic acid from simulated and actual fermentation broth. They obtained an identical total yield (65%) and a higher purity (97%) of succinic acid by using a synthetic fermentation broth rather than by using the actual fermentation broth (65 and 91%, respectively). Furthermore, Sun et al. (2019) also reported two other methods. One of these methods is sugaring-out extraction combined with crystallization for the recovery of succinic acid with a total yield of 73% and a higher purity of 98%. However, a salt-assisted technique should be used for the sugaring-out extraction of succinic acid in a t-butanol/glucose system. The drawback of this method is the high energy consumption of cyclic t-butanol utilization via vacuum distillation. The other method is ionic liquid-based sugaring-out and salting-out extraction of succinic acid with a total yield of 75% for simulated fermentation broth and a total yield of 72% for actual fermentation broth (Sun et al., 2018). These types of extraction are, however, not achieved easily in industrial production settings because the ionic liquids are expensive and cyclic utilization is complex. Lin et al. (2010) reported used resin-based vacuum distillation-crystallization and obtained succinic acid at a purity of 99% and yield of 89.5%. Furthermore, Thuy et al. (2017) applied the seeded batch cooling crystallization after pretreatment via nanofiltration and obtained succinic acid with 99% purity and 93.5% yield from high seeding experiments.

**Table 1:** Summary of succinic acid purification.

**Method of downstream process of SA**	**Product**	**Purity (%)**	**Total yield (%)**	**Solution**	**Advantage**	**Drawbacks**	**References**
Sugaring-out extraction combining crystallization	Succinic acid	98%	73%	Synthetic fermentation broth	Coupling with upstream fermentation technology	t-Butanol recovering by vacuum distillation and high energy consumption	Sun et al., 2019
Two-step membrane process	Succinic acid	85–99.5%	92%	Synthetic fermentation broth	–	–	Khunnonkwao et al., 2018
Ionic liquid-based sugaring-out and salting-out extraction	Succinic acid	– –	75%; 72%	Simulated fermentation broth; Actual fermentation broth	A sound basis for developing green, cost-effective strategies	Expensive Ionic liquids	Sun et al., 2018
Membrane separation and reactive extraction	Succinic acid	–	90%	Broth	–	Not selective enough to separate succinic acid from other acids in the broth	Prochaska et al., 2018
Pretreatment using nanofiltration Seeded batch cooling crystallization	Succinic acid	99.35%	93.47%	Fermentation broth	High purity and yield	Membrane pollution and high operation cost	Thuy et al., 2017
Salting-out extraction, vacuum distillation, crystallization, and drying; Salting-out extraction, vacuum distillation, activate carbon absorption, crystallization, and drying	Succinic acid	97%; 91%	65%; 65%	Synthetic fermentation broth; Actual fermentation broth	Low energy consumption and easy amplification	Low yield	Sun et al., 2014
Bipolar membrane electrodialysis	Succinic acid	–	90%	Synthetic broth	Suitable for continuous separation	High energy consumption, high membranes cost, and low succinate selectivity	Fu et al., 2014
Extraction	Succinic acid	–	78–85%	Fermentation broth	High output and low energy consumption	Requirement of broth pretreatment and expensive agents for reactive extraction	Kurzrock and Weuster-Botz, 2011
Centrifugation, filtration, resin-based vacuum distillation-crystallization	Succinic acid	99%	89.5%	Actual fermentation broths	High recovery yield	High energy consumption	Lin et al., 2010
One-step crystallization	Succinic acid	90%	70%	Fermentation broth	Simple process	Low succinic acid purity	Li et al., 2010
Direct vacuum distillation-crystallization	Succinic acid	97% 45%	75% 28%	Fermentation broth Synthetic broth	Easy operation and absence of additional reagents	Low succinate yield and purity, requirement of other unit operations	Luque et al., 2009
Traditional calcium precipitation coupled ion-exchange adsorption	Succinic acid	92%	52%	Fermentation broth	Easy scaling up and operation, low technical barriers, and low-priced precipitants	Water consumption, requirement of large quantities of precipitants, useless byproducts, and regeneration and cleaning of adsorbents	Li et al., 2009
Direct crystallization, co-crystallization with urea, and succinimide synthesis	Succinic acid Succinimide	99.0%	95% 80%	Fermentation broth	High Recovery, Simple Process, and low Cost	–	This study

Owing to the problems of low concentration and difficulty in recovering the remaining succinic acid from the fermentation broth after cooling crystallization, if concentrated, solvent extraction and ion exchange chromatography are used, high amounts of energy are consumed, and a great deal of wastewater is produced. Currently, research on co-crystallization is mainly focused on wastewater treatment to recover organic acids and crystalline products (Zhang et al., 2017). In this study, urea co-crystallization was used to separate succinic acid from the fermentation broth, which was achieved with a high yield, i.e., 92%.

Succinic acid must be subsequently separated from the co-crystallization of urea and succinic acid, which is a hard nut to crack. Succinic acid is usually extracted from the co-crystallization products with ether-based organic solvents and exchanged with resin. A considerable amount of energy is also required to recycle the organic solvent and water but high-value-added succinic acid derivatives, such as polybutylene succinate (Bechthold et al., 2008; Wang et al., 2014) and succinimide. According to previous reports, succinimide can be synthesized using succinic acid and urea or NH_3_ as a raw material (Fischer et al., 2011; Liu et al., 2018). Succinimide synthesis occurs by controlling the initial ratio of succinic acid to urea from the co-crystalline product of urea. In this study, the synthesis of succinimide from succinic acid and urea was investigated and the appropriate mass ratio of succinic acid to urea, reaction temperature, reaction time, amount of catalyst (phosphorous acid) were determined. The optimum reaction mass ratio of succinic acid to urea was 2:1, the most suitable amount of added phosphorous acid was 4.2% of the mass of succinic acid, and the reaction performed best at 195°C for 6 h. Finally, high-purity succinimide was obtained by recrystallization.

Urea is widely used to design and synthesize solid structures and functional materials in crystal engineering and supermolecular chemistry (Alhalaweh et al., 2010). Urea and solvents can be recycled through rectification to reduce the damage caused by solvents to the environment. Methods of synthesis involve the use of biological products as starting materials, which cause little pollution. The residual fermentation broth is harmless to organisms during preparation, and a certain amount of residual succinic acid is present in the fermentation broth. As a result, urea can be used as a biological fertilizer in the fermentation broth to promote crop growth (Shi et al., 2019). CO_2_ produced by synthesizing succinimide can be used for microbial cultivation in the pre-succinic acid fermentation (Wu et al., 2012).

## Conclusions

An efficient and environment-friendly method was developed to produce succinimide from biomass-derived succinic acid through succinic acid–urea co-crystallization. This innovative strategy could reduce production costs, recycle materials, and protect the environment from the harmful chemicals used in a conventional succinic acid purification methods. Cooling crystallization was first applied to separate the decolorized fermentation broth. The optimal conditions for cooling crystallization were determined to be 8°C, 4 h, and pH 2.0. Glucose and protein concentrations had no significant effect on the cooling crystallization process. The residue from cooling crystallization was further used for co-crystallization with urea, resulting in a recovery rate of >90% at 4°C for 12 h. This purification process was simple and cost effective. The succinimide yield could reach 80% by adjusting the mass ratio of succinic acid to urea to 2:1, adding ~4.2% solid phosphorous acid, stirring, and heating the obtained solution to 195°C via a constant temperature reaction for 6 h. This process may be remarkably advantageous in preventing environmental pollution by utilizing succinic acid for value-added chemical production and reducing the dependency on petroleum-based chemical production. In conclusion, the method established in this study not only integrates the separation of succinic acid with the synthesis of succinimide but also produced valuable intermediate products that may increase the added value of the method by improving the separation efficiency and helping the system to meet environmental requirements.

## Data Availability Statement

All datasets generated for this study are included in the article/supplementary material.

## Author Contributions

YX designed and performed all experiments under the supervision of DZ, and developed the manuscript draft. JC and ZZ supervised studies on the crystallization and co-crystallization of succinic acid from fermentation broth. BG and YW analyzed the experimental data and discussed the results with the coauthors and revised this manuscript.

### Conflict of Interest

The authors declare that the research was conducted in the absence of any commercial or financial relationships that could be construed as a potential conflict of interest.
